# Identifying malaria hotspots in Keur Soce health and demographic surveillance site in context of low transmission

**DOI:** 10.1186/1475-2875-13-453

**Published:** 2014-11-24

**Authors:** Mansour Ndiath, Babacar Faye, Badara Cisse, Jean Louis Ndiaye, Jules François Gomis, Anta Tal Dia, Oumar Gaye

**Affiliations:** Service Parasitologie, Université Cheikh Anta Diop, Dakar, Senegal; Institut de santé et de développement, UCAD, Dakar, Senegal

**Keywords:** Malaria, Hotspots, Risk factors, Prevalence, Clusters, Rapid diagnostic test

## Abstract

**Background:**

Malaria is major public health problem in Senegal. In some parts of the country, it occurs almost permanently with a seasonal increase during the rainy season. There is evidence to suggest that the prevalence of malaria in Senegal has decreased considerably during the past few years. Recent data from the Senegalese National Malaria Control Programme (NMCP) indicates that the number of malaria cases decrease from 1,500,000 in 2006 to 174,339 in 2010. With the decline of malaria morbidity in Senegal, the characterization of the new epidemiological profile of this disease is crucial for public health decision makers.

**Methods:**

SaTScan™ software using the Kulldorf method of retrospective space-time permutation and the Bernoulli purely spatial model was used to identify malaria clusters using confirmed malaria cases in 74 villages. ArcMAp was used to map malaria hotspots. Logistic regression was used to investigate risk factors for malaria hotspots in Keur Soce health and demographic surveillance site.

**Results:**

A total of 1,614 individuals in 440 randomly selected households were enrolled. The overall malaria prevalence was 12%. The malaria prevalence during the study period varied from less than 2% to more than 25% from one village to another. The results showed also that rooms located between 50 m to 100 m away from livestock holding place [adjusted O.R = 0.7, P = 0.044, 95% C.I (1.02 - 7.42)], bed net use [adjusted O.R = 1.2, P = 0.024, 95% C.I (1.02 –1.48)], are good predictors for malaria hotspots in the Keur Soce health and demographic surveillance site. The socio economic status of the household also predicted on hotspots patterns. The less poor household are 30% less likely to be classified as malaria hotspots area compared to the poorest household [adjusted O.R = 0.7, P = 0.014, 95% C.I (0.47 – 0.91)].

**Conclusion:**

The study investigated risk factors for malaria hotspots in small communities in the Keur Soce site. The result showed considerable variation of malaria prevalence between villages which cannot be detected in aggregated data. The data presented in this paper are the first step to understanding malaria in the Keur Soce site from a micro-geographic perspective.

## Background

Malaria is estimated to cause about 300 to 500 million clinical episodes causing over one million deaths annually. 60% of these episodes occur in Africa, South of the Sahara resulting in about 600,000 deaths each year [1, 2]. Most of these deaths occur in rural areas where access to drugs and prompt malaria diagnostic and treatment still remains poor. Increasing number of death attributable to malaria has continued to hamper successes made in decreasing malaria mortality among population at risk. Despite these estimates, the numbers of confirmed malaria cases had reduced up to 50% over the past decade from several high burden African countries [3], including Eritrea, Rwanda, Zanzibar [2] and Pemba [4]. This decline had changed the malaria epidemiologic profile from endemic to occurrence of clusters and or hotspots. Between 2000 and 2012, the scale-up of interventions helped to reduce malaria incidence rates by 25% globally, and by 31% in the WHO African Region [5].

In Senegal, there has recently been a sharp decline in the prevalence of malaria, coinciding with increased controls efforts, primarily the large-scale distribution of free or highly subsidized insecticide-treated bed nets [6]. According to the 2013 statistics from the Senegalese National Malaria Control Programme (NMCP), the malaria prevalence had declined within health district. The highest prevalence is localized in only 26 health district out the 76 health districts with more than 25 confirmed cases per 1,000. The Keur Soce Health and Demographic Site System (HDSS) are among those.

As prevalence has declined, the geographical distribution of malaria has become patchier, with some health posts reporting no cases during the whole year. As the malaria epidemic continues its impact in this health district, geostatistical approaches have received increasing attention as a way of determining possible “hotspots” of malaria infection and prioritizing areas for intervention. If found to exist and to have significantly excessive rates of malaria, such hotspots could be considered as surrogates for unobserved or unknown risk factor [7, 8]. However, investigating the spatial structure of the disease can be challenging. Sparsely populated, large geographical areas can mask geographical heterogeneity and may potentially cause misinterpretation of true underlying geographical patterns [9].

The Keur Soce site of the Department of Parasitology at University Cheikh Anta Diop of Dakar has been involved in many international research programme and clinical trials in malaria prevention, playing an important role in the fight against malaria infection. The role of the Department of Parasitology is essentially teaching medical doctors as well as master and PhD students. On the light of these recent changes in the epidemiological profile of the disease, it has been suggested that, control measures need to be targeted to the areas where transmission persists [10]. It is, therefore, important to be able to identify these areas, and to understand the reasons why malaria persists in some areas but virtually disappeared in others, so that rational elimination strategies can be developed.

The study investigated the geographical clustering of malaria using data from community cross sectional study during rainy season. The sample sizes were drawn from the Keur Soce HDSS database.

## Methods

### Population general information

**Figure 1:**
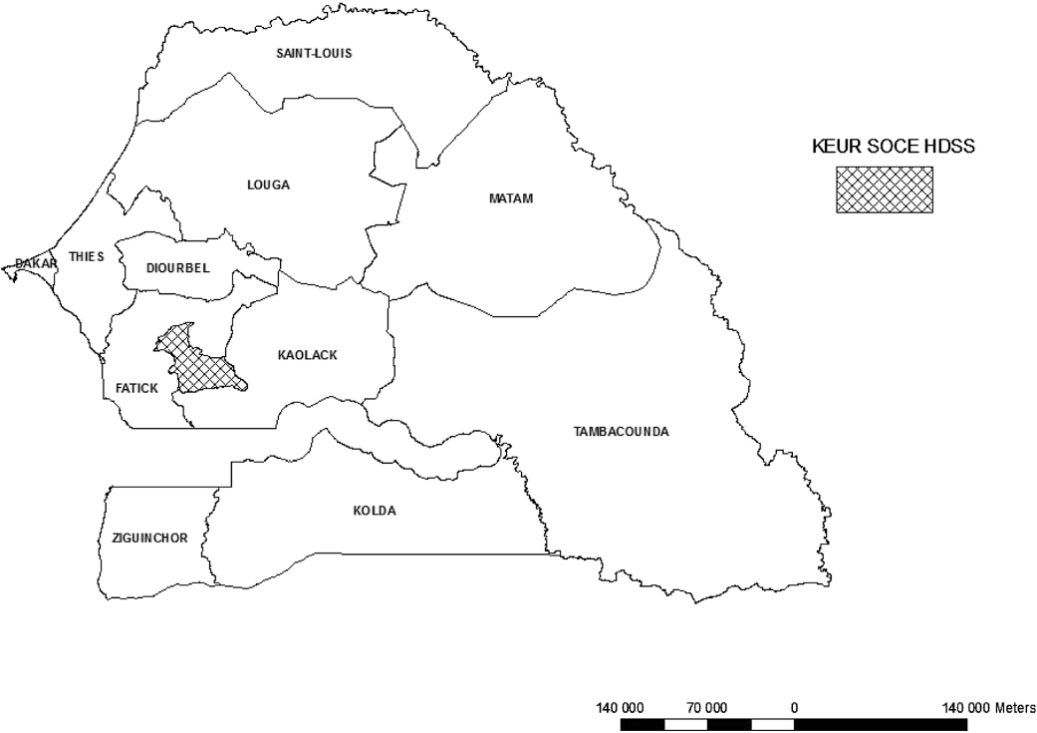
**Localization of Keur Soce HDSS in Senegal.**

The Keur Soce HDSS is being established by the Department of Parasitology University Cheikh Anta Diop (UCAD) and aim to collect data on the population’s structure, dynamics and geographical location. It is located in rural area in the region of Kaolack, in the Health District of Ndoffane. The area lies between longitudes 16°00’14.8” and 16°07’13” W and latitudes 13°51’53” and 14°00’00” N. It is located at 230 km from Dakar in the Sudano-Sahelian region of Senegal. The Keur Soce HDSS covers 74 villages (Figure 1). Regarding its localization, the site’s ecology is characterized by the alternation of a long and dry season from November to June and a short rainy season from July to October. The population of the Keur Soce HDSS as at 1st August 2013 was estimated as 32,601 inhabitants with 2371 households.

### Survey method

A community cross sectional epidemiological survey was conducted in November 2013 to assess malaria occurrence in Keur Soce HDSS. A stratified sample was done using the Keur Soce database because the knowledge attitudes practices (KAP) and socio-economic status of households may be homogeneous between villages. During the last update round, there were 2,671 households registered in the Keur Soce HDSS. Taking into account the prevalence of malaria 15% to an accuracy level of 5%, a confidence interval of 95% (α = 5%) and taking into account a cluster effect of 2, the sample was proportional to the number of households in a village. All the 74 villages were visited. The random list of the study participants was generated by stata version 12.A total of 440 households were selected and included in the study, distributed in 74 villages, including a total of 1,614 participants.

For any participant with fever or history of fever during the last 24 hours or 48 hours belonging to a selected household, a RDT and a finger prick blood sample were performed simultaneously to determine parasite carriage and a socio-economic questionnaire was used to measure household socio-economic index of the patient. Prior to the survey, field workers were trained to how to perform interview using a questionnaire, which collected information relating to the socio-economic and environmental aspects of the selected household. The questions included: use of protective measures, and the coverage of control measures in the vicinity, to understand the direct and indirect effects of these measures (ITNs being the most important); demographic and socioeconomic variables, to understand the population groups who are now at risk, proximity to mosquito breeding sites and the socio economic status of the household, cattle ownership, literacy, knowledge and awareness of malaria prevention, highest level of education attained by each participant. Malaria parasitaemia was detected by preparing blood smears in the field which were stained with 10% Giemsa. In the field, participants were tested using RDT and any positive cases were referred to the nearest health unit.

### GPS data collections

The coordinates (longitude and latitude) of all selected households (n = 440) were recorded onsite using eTrex Venture single handheld GPS receivers. GPS points were uploaded to an Access database system and cleaned for duplicates. Household with geo-coded location was checked at the field level for accuracy. Distances between points of interest were calculated using the geodist stata command [11]. Distance = √(*x*2-*x*1)^2^ + (*y2*-*y1*)^2^.

### Data analysis

Epidemiologic information and laboratory results were linked to each household's GPS data. Maps were produced with ArcMap 9.3 software. SaTScan (v. 09) was used to detect spatial clusters. Double data entry was done at informatics unit based in Dakar and data was cleaned and loaded in stata V.12 using Open Database Connectivity (ODBC).

### Spatial and temporal clusters

SaTScan™ software, version 9.1, using the Kulldorf method of retrospective space-time permutation and the Bernoulli purely spatial model [12, 13] was used to detect malaria clusters. The circular scan statistic is isotopic with respect to the rotation of the geographical area. This method has previously been validated for plotting and understanding local malaria time-space-clusters [12–16].

Observed cases in a cluster were compared to the distribution of expected cases if spatial and temporal locations of all cases were independent. The model adjusts for entirely spatial or entirely temporal clusters. With spatial adjustment, time remained dormant and during temporal analysis seasons were considered. The distribution and statistical significance of the clusters were explored by means of Monte Carlo replication of data sets under the null hypothesis with replications greater than 999 to ensure adequate power for defining clusters.

### Mapping malaria hotspots

Hierarchical techniques are like an inverted tree diagram in which two or more incidents are first grouped on the basis of some criteria (e.g. nearest neighbour). Then, the pairs are grouped into second-order clusters. The second-order clusters are then grouped into third-order clusters, and this process is repeated until either all prevalence fall into a single cluster or else the grouping criteria fails. Hotspot analysis of fixed distance conceptualization spatial relationship was performed to determine the Z score values, which determine areas of high or low clustering spatially. ArcMap version 9.3 was used to perform mapping of spatial data.

### Use of principal component analysis

Socioeconomic status was measured using an index based on ownership of assets, water and sanitation facilities, power source and housing quality and constituted the main independent variable. The assets were combined into a wealth index using weights derived through principal components analysis (PCA) using Stata 12. PCA involves breaking down assets (e.g. radio, bicycle) or household service access (e.g. water, electricity) into categorical or interval variables. The variables are then processed in order to obtain weights and principal components. The results obtained from the first principal component (explaining the most variability) are usually used to develop an index based on the formula: *A*_*j* =_*ƒ*_1_*x* (*a*_*ji* -_*a*_*i*)_/(*S*_1_)_+ … … …. +_*ƒ*_*N*_*x*(*aj*_*N* -_*a*_*N*)_/(*S*_*N*_) [17]. Where f_1_ is the scoring factor or weights for the first asset (or service), and a_1_ and s_1_ are the mean and the standard deviation of the first assets (or service) variable over all households respectively. Statistical analyses were done with Stata version 12 software. Multiple logistic regression models were used to examine the effects of socio-economic and environmental risk factors for residing in hotspot areas.

### Ethics statement

The study protocol was approved by the Senegalese Ministry of Health through the National Ethics Committee. One day before the survey, a field worker visited each household to provide explanations concerning the study. All participants signed an informed consent before being enrolled in the study and handled a signed or marked with a fingerprint informed consent. All household heads signed the consent form. Participants found to be positive for *Plasmodium falciparum* infection were automatically referred to the nearest health unit.

## Results

### Malaria prevalence

**Figure 2:**
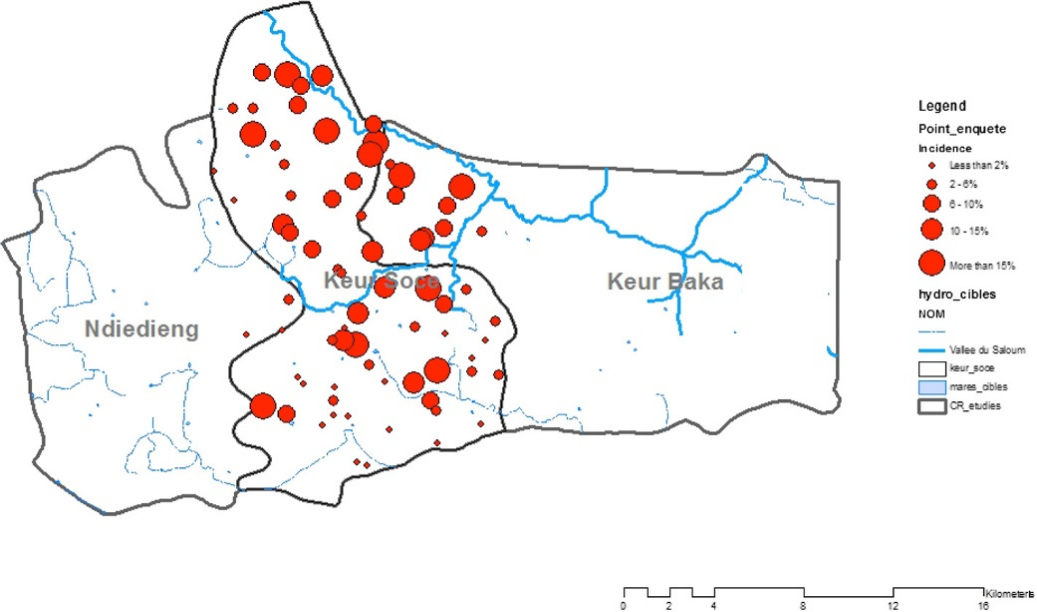
**Prevalence of malaria in Keur Soce.**

A total of 1,614 individuals from 446 households were included in the analysis. The overall malaria prevalence was 12%. The malaria prevalence during the study period varied from less than 2% to more than 25% from one village to another. The highest malaria prevalence rates were identified in north of the Keur Soce HDSS (Figure 2). Most of these villages are located near the wet area. Table 1 describes the participants and theirs household characteristics. Most the study participants were female (66.04%) and most of them were more than 10 years of age (49.75%). Fifty-tree percent of them had never been at formal school. The household size varied from less than five persons (20.13%) to more than 10 persons (19.02%). Sixty six percent of household members declared having bed nets. The main drinking water source is water from the well (57.18%) and most toilet facilities are latrines (57.31%).

**Table 1:** **Individual and household characteristics of study participants**

Characteristics	Categories	Number	Percent
Gender	Male	584	36.18
	Female	1066	66.04
Age	Less than 5 years	563	34.88
	5 – 10 years	236	14.62
	More than 10 years	803	49.75
Marital status	Unmarried	1020	63.19
	Married	594	36.80
	None	856	53.03
Education	Primary	613	37.98
	Secondary	145	8.98
	Less than 5 persons	325	20.13
Household size	5 – 10 persons	982	60.84
	More than 10 persons	307	19.02
Bed net ownership	Yes	1066	66.04
	No	548	33.95
	Pipped water	294	18.21
Water source	Well water	923	57.18
	Others	629	39.97
	Flush	237	14.68
Toilet facilities	Latrine	925	57.31
	Others	452	28.00
Lighting source	Electricity	1032	63.94
	Solar	245	15.17
	Others	337	20.87
Cooking fuel	Gas	214	13.2
	Charcoal	400	24.78
	Firewood	774	47.95
	Others	226	14.00

### Spatial cluster of malaria

**Figure 3:**
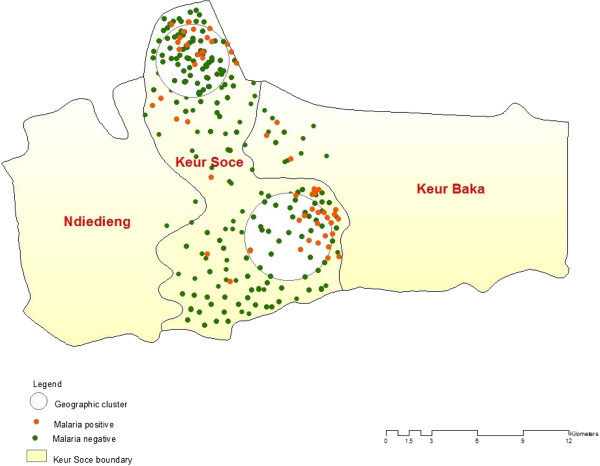
**Localization of malaria hotspots in Keur Soce.**

SaTScan was used to detect malaria clusters in Keur Soce HDSS (n = 1614). Table 2 shows that there were four clusters in total. Among them, one was most likely clustered and three were secondary clusters. The first cluster is located in the north and included villages such as Sama Toucouleur, Keur Moussa Ly, Fass guéladio and Fass Toucouleur (Figure 3). These villages presented the highest prevalence rate with more than 25%. According to the analysis from Satscan theses villages had high risk of being classified as a malaria cluster (RR = 7.60; P = 0.002). The second cluster is localized south of Keur Soce HDSS. The second cluster included Kacothie, Keur Birame Diouma, and Keur Makam (RR = 2.74; P = 0.0084). Information on the spatial distribution of populations and services is essential to understand access to health services. There should be specially focused strategies to optimize health care for people living in the high-risk areas. Spatial analysis is an important tool for monitoring the malaria occurrence, predicting future treatment demands, and targeting areas for public health interventions.

**Table 2:** **Spatial malaria clusters in Keur Soce detected by SaTScan v9.0.2**

Clusters	Population	No of cases	Expected cases	Relative risk	Log likelihood ratio	P_value
**Most likely cluster**						
	92	28	19.92	7.60	15.606779	0.0012
**Secondary clusters**						
	60	15	14.63	2.74	9.296210	0.0084
	9	7	3.22	4.33	6.614584	0.918
	6	6	1.06	4.06	2.473340	0.562

### Risk factors malaria hotspots

Table 3 summarizes individual and household level risk factors. Logistic regression was used to investigate the effect of socio-economic and environment risk factors of residing in a hotspots area. The findings show an increase risks of residing in hotspots among the older individuals compared to the younger children. The individuals aged five to nine years were 50% more likely to be residing in the hotspots area compared to those less than five years of age [crude O.R = 1.5, P = 0.026, 95% C.I (1.05 - 2.34)]; while adjusted with other factors, this increased the risks four times [adjusted O.R = 4.7, P = 0.027, 95% C.I (2.85 - 5.26)]. The sleeping rooms made with Banco are 80% more like to be included in the hotspots compared to those made in cement [adjusted O.R = 1.8, P = 0.061, 95% C.I (0.97 - 3.64 )] those also made with thatch are also two times more likely to be considered in the hotpots area [adjusted O.R = 2.2, P = 0.016, 95% C.I (1.16 - 4.35 )].

**Table 3:** **Risk factors for malaria hotspots**

Factors	Univariate analysis	Multivariate analysis
	OR (95% CI) P	OR (95% CI) P
**Age group**		
Less than 5 years	1	1
5 – 9 years	1.5 (1.05 - 2.34) 0.026	4.7 (2.85 - 5.26) 0.027
More than 9 years	1.4 (0.97 - 2.22) 0.062	2.4 (1.67 - 3.81) 0.038
**Materials of sleeping rooms**		
Cement	1	1
Banco	1.5 (1.10 - 2.31) 0.014	1.8 (0.97 - 3.64) 0.061
Thatch	1.5 (0.90 - 2.36) 0.070	2.2 (1.16 - 4.35) 0.016
**Materials of floor**		
Cement	1	1
Banco	1.9 (1.31 - 2.75) 0.001	0.9 (0.83 - 1.12) 0.646
Other	1.3 (0.85 - 2.27) 0.182	0.4 (0.29 - 0.64) 0.000
**Rooms having window**		
Yes	1	1
No	1.6 (1.01 - 2.82) 0.045	1.0 (0.92 - 1.10) 0.799
**Distance to livestock places**		
Less than 50 m	1	1
50 – 100 m	0.4 (0.25 - 0.81) 0.009	0.7 (1.02 - 7.42) 0.044
More than 100 m	1.6 (0.74 - 1.71) 0.577	2.1 (1.15 - 8.31) 0.025
**Bednet use**		
Yes	1	1
No	3.9 (1.39 - 6.22) 0.002	1.2 (1.02 - 1.48) 0.024
**Goat ownership**		
Yes	1	1
No	1.5 (1.09 - 2.10) 0.012	1.0 (0.99 - 1.12) 0.070
**SES of household**		
Poorest	1	1
Poorer	0.5 (0.26 - 1.32) 0.202	0.8 (0.63 - 1.08) 0.175
Poor	0.8 (0.38 - 1.81) 0.646	0.9 (0.76 - 1.27) 0.921
Less poor	2.4 (1.14 - 5.08) 0.021	0.7 (0.47 - 0.91) 0.014
Least poor	1.6 (0.76 - 3.37) 0.215	0.6 (0.45 - 0.94) 0.024

The results showed also that rooms located between 50 m to 100 m away from livestock [adjusted O.R = 0.7, P = 0.044, 95% C.I (1.02 - 7.42)], bed net use [adjusted O.R = 1.2, P = 0.024, 95% C.I (1.02 –1.48)] are good predictors for malaria hotspots in Keur Soce HDSS. The socio-economic status of the household also predicted hotspots patterns. The less poor households were 30% less likely to be classified as malaria hotspots area compared to the poorest households [adjusted O.R = 0.7, P = 0.014, 95% C.I (0.47 – 0.91)]. High coverage of bed nets was an important finding in this study. This was associated with the likelihood of malaria hotspots occurrence in the Keur Soce.

## Discussion

This study shows that malaria prevalence varied from less than 2% to 25% in Keur Soce. The highest malaria prevalence is located north and south of the study area. The Spatial Scan Statistics program was used to investigate geographical patterns and variations of malaria prevalence within the relatively homogeneous population. Strong statistical evidence of malaria clustering in the Keur Soce communities was found. The RR and corresponding p-value for most of the clusters are very significant so there is little doubt that these are high-risk areas. This result supports the notion that risk factors for malaria might be associated with certain specific socio-economic characteristics, which could be targeted to improve existing public health prevention measures aimed at the general population.

Prevalence of malaria in Senegal has always been reported either on a national basis or a regional level [18–20]. While it is important and necessary to report these figures at community or village level, such aggregated data at national and regional level may mask the spatial heterogeneity of the malaria prevalence. Hence, national level prevalence rates may not reveal the full impact of the epidemic on different geographical regions. This is particularly urgent and necessary in a Health district such as Keur Soce, where malaria prevalence is still high.

The results from this study support the conclusion that risks for malaria hotspots are associated with definable socio-demographic factors, which may be fundamental ecological units of malaria transmission [3, 21, 22]. A multitude of other factors may have an impact in these mostly rural or peri-rural settings, creating a context in which the impact of geographical factors and knowledge, attitudes and behaviours on malaria prevalence and incidence may be particularly relevant. Distance to the nearest breeding site [23–26], walling material [27], and bed net coverage [24] were independent predictors of living in a hot spot of malaria transmission. These results may be due to fundamental differences between the communities with regard to health care centres, population density and other socio-economic factors. These data provide new evidence to support the need to investigate potential sources of infection and to study transmission patterns in the community in order to apply relevant interventions for prevention of this devastating disease.

Socio-economic status of households was an important risk factor for malaria in the study area, where households of lower to moderate socio-economic status were at a more than 70% higher risk compared to households of highest socio-economic status. Similar findings have been reported elsewhere [28–30]. Poor housing was also among the risk factors for malaria in the study area, whereby individuals living in houses built with banco and thatch were at a higher risk compared to individuals of similar socio-economic status but living in better houses. Similar findings were reported by a study in Sri Lanka where mosquito abundance was associated with the poor housing construction [31] and in South Africa where a case–control study showed a six fold risk of malaria in houses built of mud walls compared to bricks [29]. This indicates that better houses do not give easy entry and hiding places for mosquitoes [32].

## Conclusion

This community cross sectional study in a small geographic provided some interesting findings about identification and characterization of malaria hotspots in Senegalese rural area. The result showed considerable variation of malaria prevalence between villages which cannot be detected in aggregated data. The methodology used for identifying clusters could be a useful tool for detecting hotspots in other part of the region. The bed net use and goat ownership are independent and important factors for identification of malaria hotspots in Keur Soce health and demographic surveillance site. This study provides a first attempt to visually and quantitatively describe the geographical characteristics of malaria hotspots in an area where the disease is known to be declining considerably. The results from the study showed that with malaria decline, intervention and treatment strategies may need to be adapted to the context of malaria hotspots.
